# Hypothermic circulatory arrest induced coagulopathy: rotational thromboelastometry analysis

**DOI:** 10.1007/s11748-020-01399-y

**Published:** 2020-06-07

**Authors:** Hayato Ise, Hiroto Kitahara, Kyohei Oyama, Keiya Takahashi, Hirotsugu Kanda, Satoshi Fujii, Takayuki Kunisawa, Hiroyuki Kamiya

**Affiliations:** 1 Department of Cardiac Surgery, Asahikawa Medical University, Midorigaoka-Higashi 2-1-1-1, Asahikawa, Hokkaido 078-8510 Japan; 2 Department of Anesthesiology, Asahikawa Medical University, Asahikawa, Hokkaido Japan; 3 Department of Laboratory Medicine, Asahikawa Medical University, Asahikawa, Hokkaido Japan

**Keywords:** Hypothermic circulatory arrest, Coagulopathy, Rotational thromboelastometry, Maximum clot elasticity

## Abstract

**Objectives:**

Hypothermic circulatory arrest (HCA) has been considered to cause coagulopathy during cardiac surgery. However, coagulopathy associated with HCA has not been understood clearly in details. The objective of this study is to analyze the details of coagulopathy related to HCA in cardiac surgery by using rotational thromboelastometry (ROTEM).

**Methods:**

We retrospectively analyzed 38 patients who underwent elective cardiac surgery (HCA group = 12, non-HCA group = 26) in our hospital. Blood samples were collected before and after cardiopulmonary bypass (CPB). Standard laboratory tests (SLTs) and ROTEM were performed. We performed four ROTEM assays (EXTEM, INTEM, HEPTEM and FIBTEM) and analyzed the following ROTEM parameters: clotting time (CT), clot formation time (CFT), maximum clot firmness (MCF) and maximum clot elasticity (MCE). The amount of perioperative bleeding, intraoperative transfusion and perioperative data were compared between the HCA and non-HCA group.

**Results:**

Operation time and hemostatic time were significantly longer in the HCA group, whereas CPB time had no difference between the groups. The amount of perioperative bleeding and intraoperative transfusion were much higher in the HCA group. SLTs showed no difference between the groups both after anesthesia induction and after protamine reversal. In ROTEM analysis, MCE contributed by platelet was reduced in the HCA group, whereas MCE contributed by fibrinogen had no difference.

**Conclusion:**

Our study confirmed that the amount of perioperative bleeding and intraoperative transfusion were significantly higher in the HCA group. ROTEM analysis would indicate that clot firmness contributed by platelet component is reduced by HCA in cardiac surgery.

## Introduction

Hypothermic circulatory arrest (HCA) is an effective procedure to perform open anastomosis in bloodless fields while protecting whole organs in aortic surgery [1–3]. It is well-known that hypothermia may cause coagulopathy, but it is difficult to prove in clinical practice [3, 4]. Perioperative standard laboratory tests (SLTs) of coagulation parameters including fibrinogen, prothrombin time (PT), PT international normalized ratio (PT-INR) and activated partial thromboplastin time (aPTT) are commonly used to detect severity of coagulopathy. However, it is unclear that those tests precisely reflect coagulopathy because they are based on only plasma [5]. Previous studies described that hypothermia caused the slowing down of enzyme processes in the coagulation cascade, disruption of platelet function and disordered fibrinolysis [6]. SLTs do not give the information of blood coagulation and fibrinolysis, so the details of coagulopathy associated with HCA during cardiac surgery has been poorly understood until now.

Rotational thromboelastometry (ROTEM) is one of a point-of-care coagulation tests with whole blood viscoelastic hemostatic assays (VHA), which can rapidly assess the detail of coagulation profiles including clot initiation, clot formation, clot stability and fibrinolysis [7]. Thus, ROTEM is an appropriate method to clarify the details of coagulopathy associated with HCA. However, there have been few studies analyzing coagulopathy associated with HCA by using ROTEM [8].

The objective of this observational study was to analyze coagulopathy in cardiac surgery with or without HCA, using not only SLTs but also ROTEM, and to compare the amount of perioperative bleeding and intraoperative transfusion of blood products.

## Materials and methods

### Patient population

We retrospectively investigated 89 patients who underwent cardiac surgery with cardiopulmonary bypass (CPB) in our hospital from January 2016 to December 2016. Among them, 38 patients who underwent cardiac surgery with or without HCA were reviewed. The cohorts were divided into two groups; 12 patients in the HCA group and 26 patients in the non-HCA group. ROTEM analysis was routinely performed in our institute in cardiac surgery. Both SLTs and ROTEM analysis were performed simultaneously before and after initiation of CPB. Minimally invasive cardiac surgery (*n* = 16), acute aortic dissection (*n* = 1) and ventricular septal defect (*n* = 2) were excluded. Other exclusion criteria were incomplete ROTEM (*n* = 16), SLTs (*n* = 14), or both of them (*n* = 2). All 38 cases were electively performed with median sternotomy approach. Institutional Review Board of Asahikawa Medical University approved this retrospective observational study (no. 17068).

### Intraoperative management

Our institutional standard anesthetic management was used to all patients. Before CPB, heparin (300 U/kg) was administrated to maintain activated clotting time (ACT) more than 480 s, and it was maintained during CPB. HCA was started when the rectal temperature reached mild to deep hypothermia (26 ± 0.4 °C). After weaning of CPB, heparin was reversed by protamine (3 mg/kg). After protamine administration, transfusion of blood products was started. Packed red blood cells were transfused when hemoglobin (Hb) level was less than 8 g/dL, and fresh frozen plasma was transfused when prolonged aPTT or PT-INR were detected. Platelet concentrate (PC) was transfused when platelet count (Plt) was less than 8 × 10^9^/L. Cryoprecipitate was given when fibrinogen level was less than 1.5 g/L. Fibrinogen concentrates were administrated when fibrinogen level was still less than 1.5 g/L after administration of cryoprecipitate. Recombinant factor VIIa was administrated when excessive bleeding continued regardless of transfusion of blood products, cryoprecipitate and fibrinogen concentrate. Hemostatic time, which was defined as time from protamine reversal to end of the operation, was measured.

### Blood sampling

Blood samples were taken at 4 time points: after anesthesia induction (S-1), during CPB, 5 min after protamine reversal (S-2) and 1 h after operation. SLTs were performed at all the 4 points, which included Hb, Plt, APTT, PT-INR, fibrinogen and antithrombin (AT3). ROTEM analysis was performed at the 2 points, after anesthesia induction (R-1) and protamine reversal (R-2).

### ROTEM measurements

**Fig. 1:**
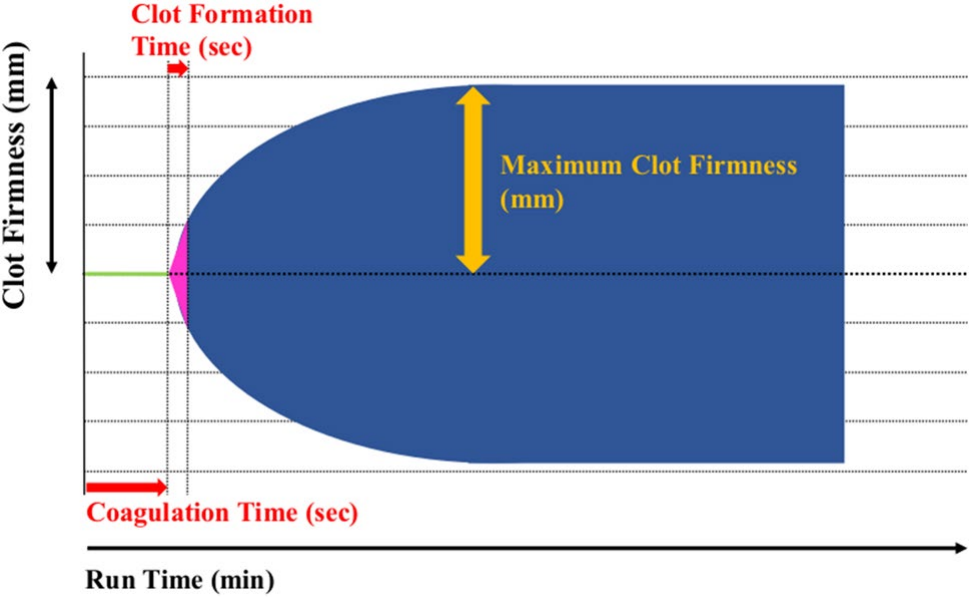
The tracing indicted a typical ROTEM result

**Fig. 2:**
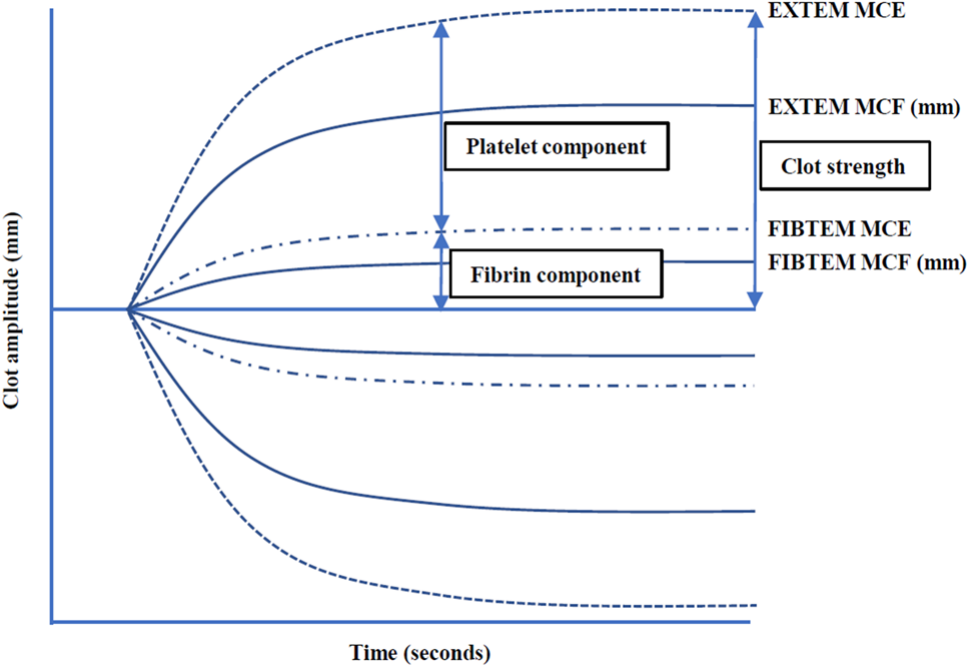
The graph described platelet and fibrin contribution to actual clot strength in thromboelastometry. MCE was calculated from MCF. MCE = (100 × MCF)/(100 − MCF). *MCF* maximum clot firmness, *MCE* maximum clot elasticity

ROTEM analyses were performed with ROTEM delta (Tem Systems Inc., Munich, Germany) according to the manufacturer’s instruction. EXTEM, INTEM, FIBTEM and HEPTEM assays were performed. EXTEM assay shows clot strength with extrinsic pathway activation of whole blood with tissue factor. FIBTEM assay also indicates clot strength but platelet contribution to clot strength is prevented by addition of cytochalasin D. INTEM assay shows intrinsic pathway. HEPTEM assay is similar to INTEM assay but it contains heparinase to inhibit heparin. Figure 1 shows a typical ROTEM tracing result. Clotting time (CT) reflecting initiation of the clotting cascade and clot formation time (CFT) reflecting fibrin polymerization and stabilization of the clot were measured in EXTEM and INTEM assays. Maximum clot firmness (MCF), which indicating clot strength, was measured in EXTEM, INTEM and FIBTEM assays. In HEPTEM assay, INTEM-HEPTEM-CT was calculated as HEPTEM-CT subtracted from INTEM-CT. INTEM-HEPTEM-CT reflects the influence of residual heparin. The maximum clot elasticity (MCE), which reflects actual physical properties of clot strength (Fig. 2), was calculated to accommodate Hook’s law [9, 10]. MCE platelets, which reflects actual platelet contribution to clot strength, was calculated as FIBTEM-MCE subtracted from EXTEM-MCE [9, 10]. Clot elasticity is considered to reflect actual force with which the clot resists the rotation in the ROTEM device, whereas clot amplitude is contributed not only by clot elasticity but also by clot viscosity. Furthermore, the relationship between clot elasticity and clot amplitude is nonlinear. Therefore, Solomon et al. reported that clot amplitude is not suitable parameter for the estimation of the platelet and fibrin contribution to the clot strength [8–10].

### Statistical analysis

All statistical data were analyzed by using SPSS 26.0 for Windows (IBM Corp., Armonk, NY, US). Values are described as mean ± SD for continuous data with a normal distribution and count or percent for categorical variables. Fischer’s exact test was used for categorical variables. Wilcoxon signed rank test was used to compare continuous variables between the two time points (S-1 vs S-2, R-1 vs R-2) in each group. Mann–Whitney *U* test was used to compare continuous variables between the two groups. Statistical significance was defined as *P* < 0.05.

## Results

### Patient characteristics

All patient characteristics and surgical procedures are described in Table 1. There were no significant differences between the two groups regarding age, sex, and body mass index. All of the HCA group had aortic surgeries for aortic aneurysm, and a few cases of concomitant valve surgery and coronary artery bypass grafting (CABG) were performed. In most of the non-HCA group, valve surgeries were performed. Concomitant procedures in the non-HCA group were mainly CABG. Two aortic cases in the non-HCA group were aortic root surgery.

**Table 1:** Characteristics and data of the groups

	HCA (+)	HCA (−)	*P* value
Number of patients (*n*)	12	26	
Age (years)	72.9 ± 1.5	73.2 ± 1.6	0.25
Sex (M/F)	6/6	14/12	1.0
BMI (kg/m^2^)	22.4 ± 1.1	22.1 ± 0.7	0.41
Surgical procedures (*n*)			
Aorta	12	2	
TAR	9	0	
Hemiarch	3	0	
Root	1	2	
Valve	3	24	
Single	3 (A:3)	15 (A:13 M:2)	
Double	0	7 (A + M:2, A + T:1, M + T:4)	
Triple	0	2	
CABG	2	14	
Bypass (*n*)			
1	1	4	
2	1	5	
3	0	5	

*BMI* body mass index, *TAR* total arch replacement, *CABG* coronary artery bypass grafting, *A* aortic valve, *M* mitral valve, *T* tricuspid valve

### Perioperative data

Perioperative data are shown in Table 2. The lowest temperature during operation was about 8 °C lower in the HCA group compared with the non-HCA group. There were no significant differences in CPB time and Aortic cross clamp time between the two groups, but the total operation time and hemostatic time in the HCA group were significantly longer than the non-HCA group. The volume of perioperative bleeding and intraoperative transfusion of blood products were significantly higher in the HCA group. The HCA group required more cryoprecipitate use compared with the non-HCA group.

**Table 2:** Perioperative data of the groups

	HCA (+)	HCA (−)	*P* value
Operation time (min)	404.4 ± 30.7	328.3 ± 20.0	0.049
CPB time (min)	182.5 ± 18.2	158.5 ± 11.2	0.41
Aortic cross clamp time (min)	110.5 ± 13.5	124.4 ± 9.2	0.29
Circulatory arrest time (min)	40.3 ± 4.3	0	< 0.01
Hemostatic time (min)	157.8 ± 19.2	77.6 ± 11.6	< 0.01
Lowest body temperature (°C)	26.3 ± 0.5	34.0 ± 0.1	< 0.01
Bleeding volume intra-OP (ml)	5263 ± 1081	1811 ± 377	< 0.01
Bleeding volume until 12 h (ml)	1144 ± 198	763 ± 100	0.042
PRBC intra-OP (unit)	12.6 ± 2.6	7.3 ± 1.4	0.021
FFP intra-OP (unit)	34.1 ± 4.6	13.7 ± 2.6	< 0.01
PC intra-OP (unit)	39.2 ± 3.7	20.2 ± 3.8	< 0.01
Patients treated with			
Cryoprecipitate (*n*, %)	10 (83.3)	7 (26.9)	< 0.01
Fibrinogen (*n*, %)	2 (16.6)	1 (3.8)	0.23
rVIIa (*n*, %)	2 (16.6)	3 (11.5)	0.64

*CPB* cardiopulmonary bypass, *OP* operation, *PRBC* packed red blood cells, *FFP* fresh frozen plasma, *PC* platelet concentrates

### SLTs and ROTEM analyses

**Fig. 3:**
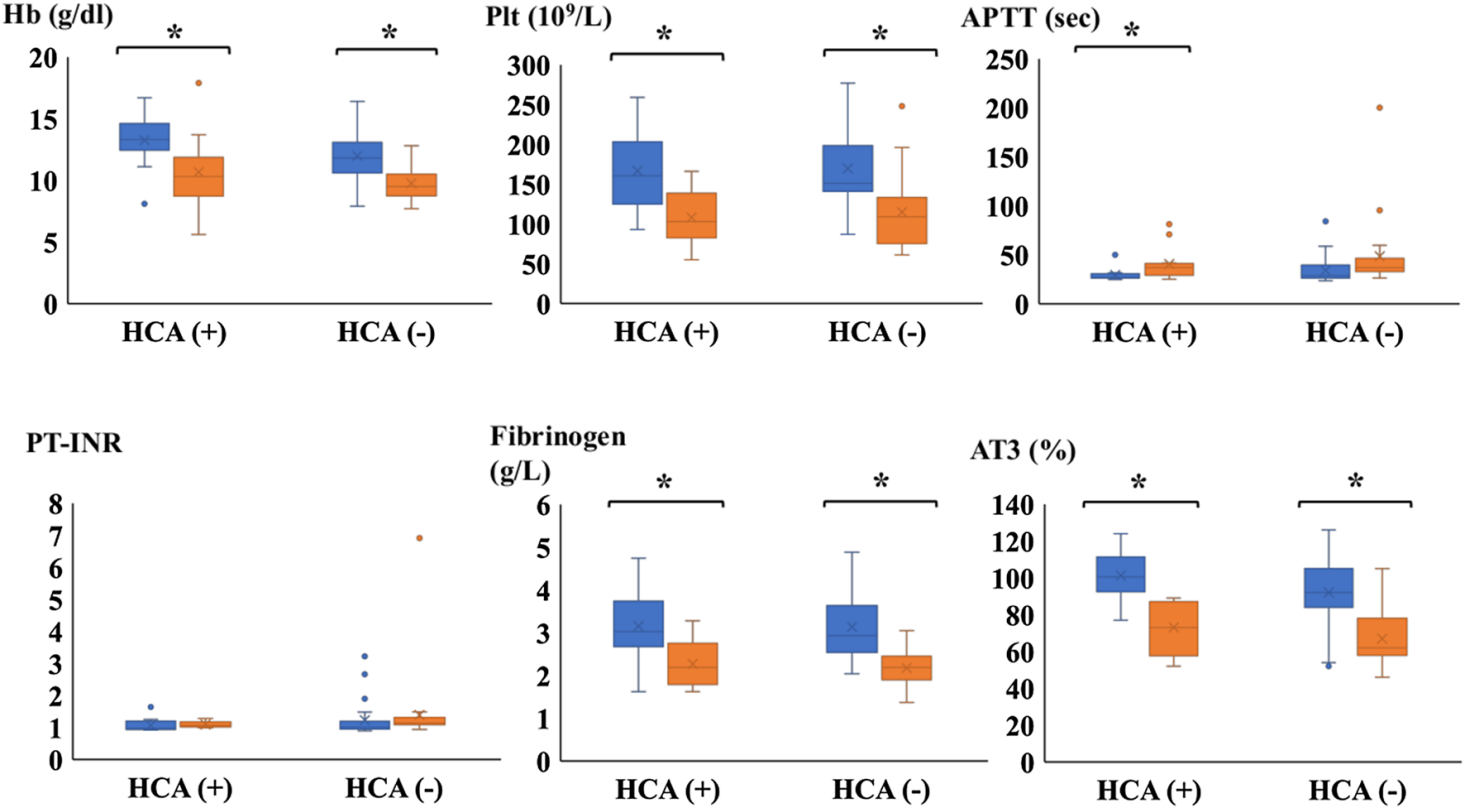
The graph indicated parameters of SLTs at two time points (S-1 and S-2). Blue box plot described S-1, orange box plot described S-2. Error bars represents SD. Asterisks represented *p* < 0.05 between S-1 and S-2. *Hb* hemoglobin, *Plt* platelet, *APTT* activated partial thromboplastin time, *PT-INR* prothrombin time international normalized ratio; *AT3* antithrombin 3. *SLTSs* standard laboratory tests, *S-1* SLTs after anesthesia induction, *S-2* SLTs after protamine reversal

**Fig. 4:**
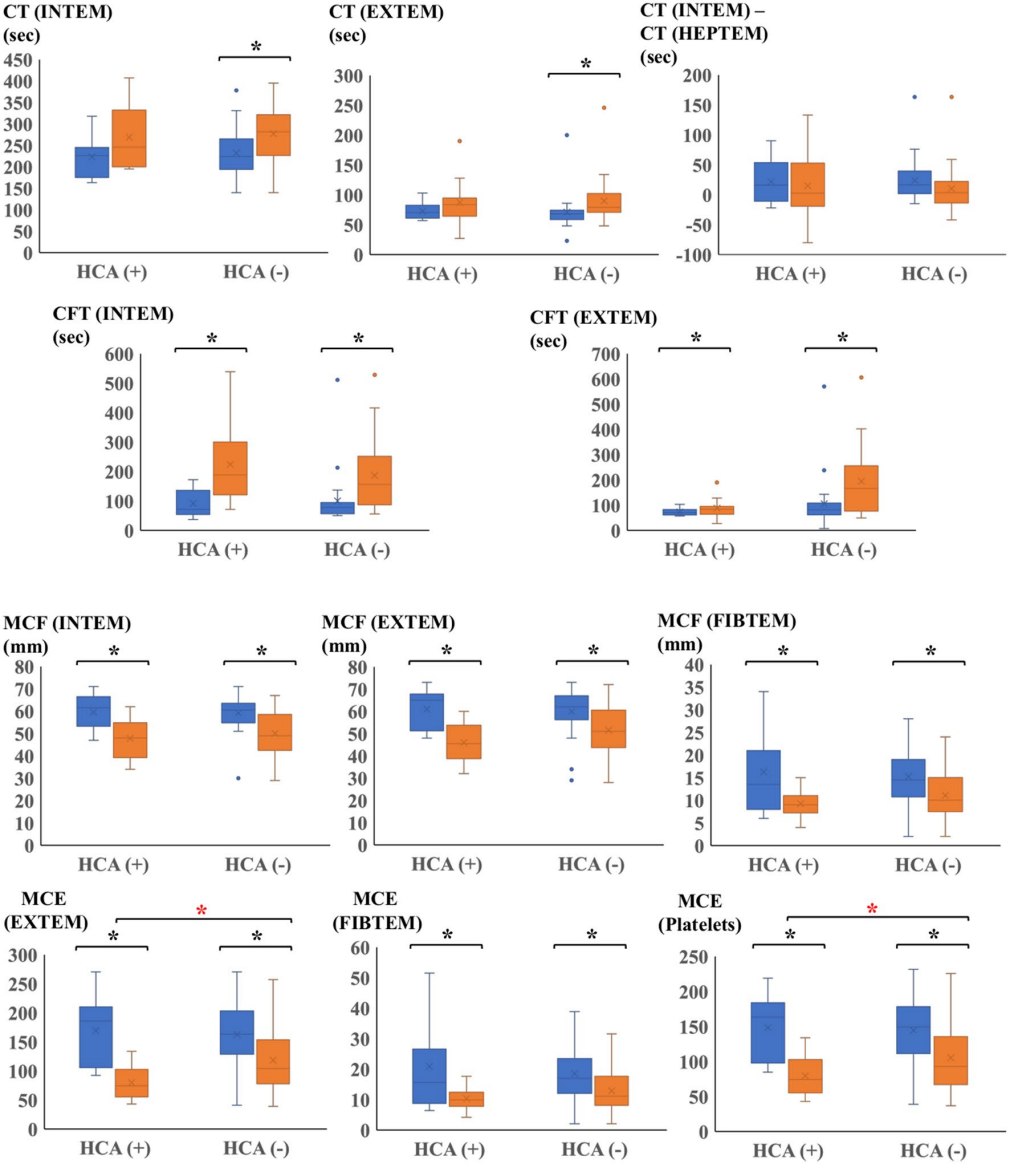
The graph indicated parameters of ROTEM at two time points (R-1 and R-2). Blue box plot described R-1, orange box plot described R-2. Error bars represents SD. Asterisks represented *p* < 0.05 between S-1 and S-2. Red asterisks represented *p* < 0.05 between R-2 of the HCA group and R-2 of the non-HCA group. *CT* clotting time, *CFT* clot formation time, *MCF* maximum clot firmness, *MCE* maximum clot elasticity, *R-1* ROTEM after anesthesia induction, *R-2* ROTEM after protamine reversal

The results of SLTs after anesthesia induction (S-1) and after protamine reversal (S-2) are shown in Fig. 3. Hb, Plt, Fib and AT3 decreased significantly at S-2 in each group. APTT was significantly extended at S-2 only in the HCA group. However, there were no significant differences in SLTs between the groups at both time points. The results of ROTEM after anesthesia induction (R-1) and after protamine reversal (R-2) are shown in Fig. 4. In the non-HCA group, both INTEM CT and EXTEM CT were significantly extended at R-2. There was no significant difference in INTEM-HEPTEM-CT between the two groups at R-2. It revealed that there was no difference in the effect of residual heparin between the groups. EXTEM CFT and INTEM CFT decreased significantly at R-2 in each group, though they were similar between the groups at both time points. EXTEM MCF and FIBTEM MCF were decreased in the both groups at R-2, but there was no significant difference between the groups. It indicates that EXTEM MCF and FIBTEM MCF were not predictive indicators of bleeding. In contrast, EXTEM-MCE was significantly lower at R-2 in the HCA group compared to the non-HCA group, meaning that clot strength was weaker in the HCA group after the end of CPB. FIBTEM-MCE was similar at R-2 between the groups and MCE platelet was significantly lower in the HCA group at R-2 compared to the non-HCA group. It indicated that clot strength was weaker in the HCA group not because of fibrinogen level but because of platelet components.

## Discussion

To our knowledge, few studies have analyzed coagulopathy associated with HCA in detail by using ROTEM [8]. The principal findings of our present study are as follows: First, the amount of bleeding and transfusion in cardiac surgery with HCA was significantly higher than that without HCA. Second, ROTEM analysis showed the significant decrease of clot strength contributed by platelet component in patients who underwent cardiac surgery with HCA.

This study confirmed that the amount of bleeding and transfusion use in cardiac surgery with HCA were much higher than that without HCA. It is well-known fact that both CPB and HCA are one of the main causes of coagulopathy in cardiac surgery. During CPB, activation and dysfunction of intrinsic and extrinsic pathway, platelet dysfunction and excessive anticoagulation by heparin infusion could lead to excessive bleeding [2, 4, 9]. In our study, CPB time was not different between the groups, but the operation time and hemostatic time in the HCA group were significantly longer than the non-HCA group. It might mean that coagulopathy got worse by HCA but CPB time in this study. However, it is difficult to understand the details of coagulopathy during cardiac surgery with HCA by only using SLTs, because it takes much time to get results and not actually reflects coagulopathy in the whole blood. Our results showed the limitation of SLTs for assessment of coagulopathy during cardiac surgery. In this study, all parameters of SLTs were not significantly different between the groups at both time points. It seems that SLTs do not reflect actual coagulopathy during surgery because they are based only on plasma. Especially in cardiac surgery, status of coagulation and fibrinolysis is dramatically changing with time, due to administration of heparin, CPB, body temperature change and administration of protamine. Therefore, it is important to assess the actual balance of coagulation and fibrinolysis at real time with whole blood to reduce intraoperative bleeding and transfusion use [11].

ROTEM analysis in this study indicated that excessive bleeding in cardiac surgery with HCA might be mainly caused by decrease of clot firmness contributed by platelet components. ROTEM is one of VHA assays using whole blood sample and rapidly provides information about quality and dynamics of clotting cascade. ROTEM is often used in cardiac surgery, and its benefit in reducing perioperative transfusion use in cardiac surgery has been described [5, 11]. FIBTEM MCF is commonly used as a sensitive predictor of hypofibrinogenemia. Administration of fibrinogen concentrate guided by FIBTEM MCF has been reported to be beneficial for reduction of transfusion use in previous studies [12–14]. However, some recent other double-blinded and placebo-controlled randomized trials failed to reveal the effect of reducing transfusion use by FIBTEM MCF guided administration of fibrinogen concentrates in cardiac surgery [15, 16]. In our study, the levels of FIBTEM-MCF were within normal range and similar between the groups, though the amount of bleeding and transfusion use were much higher in the HCA group. It indicates that fibrinogen is not precisely predicting intraoperative bleeding or transfusion use [17, 18].

Correlations between bleeding and platelet counts and aggregations are well-known [19]. In our study, we calculated and analyzed MCE platelet component in ROTEM, which reflects actual clot strength contributed by platelets. MCF is commonly used as a parameter of clot strength. On the other hand, Solomon et al. pointed out that MCF was inappropriate as a parameter because clot amplitude and clot elasticity was not properly correlated, therefore they recommended to use MCE as a parameter of clot strength [8]. Their study indicated that platelet contribution to clot strength was underestimated by using MCF. In our study, MCE platelets after protamine reversal in the HCA group was significantly lower than the non-HCA group. It indicates that contribution of platelet component to clot strength in the HCA group decreased. This finding suggests that we can possibly use MCE platelets as a parameter to decide administration of PC during surgery.

Lang et al. reported that clot strength, expressed as MCE, increased when platelet count changed from 10^9^/L to 10 × 10^9^/L in normal fibrinogen level (1.5–3.5 g/L), then clot strength tended to reach plateau in normal range of platelet count [20]. They also reported that clot strength increased in fibrinogen-dependent manner irrespective of platelet count. In low platelet count condition, abundant GPIIb/IIIa receptors expressed and captured fibrinogen, so interactions between fibrinogen and platelet increased in fibrinogen-dependent manner. Many interactions between platelet and fibrinogen complements clot firmness. Their study indicates that adequate fibrinogen level is needed in low platelet condition (< 10 × 10^9^/L) and low platelet count causes inadequate clot strength in normal fibrinogen level. In our study, fibrinogen level in the HCA group was higher than the non-HCA group at both time points, but clot strength was not enough to prevent excessive bleeding. It might be because of low platelet counts (< 10 × 10^9^/L) or platelet dysfunction. In such case, administration of much higher dose of fibrinogen concentrate or administration of PC might be needed. Our study indicated that not only adequate fibrinogen level but also platelet counts are important to make firmer clot formation. In ROTEM analysis, MCE platelets and FIBTEM MCE were useful parameters to see actual clot strength contributed by both platelet and fibrinogen. Further investigations are needed to detect the cut off value of the parameters to decide the need of PC and fibrinogen concentrate during surgery.

## Limitations

This is a retrospective observational analysis of small sample size. Due to observational nature of the study, the sample size of the HCA group was smaller and the surgical procedures between the groups were different. The different sample size in the two groups limited the power of ROTEM analysis results between the groups. The number of aortic surgeries was smaller in the HCA group, which might affect the amount of intraoperative bleeding and transfusion use. We use fibrin sealant patches at anastomosis site more often in aortic surgery, so it might have some effects on hemostatic time or bleeding volume. MCE platelets, which calculated by EXTEM MCE and FIBTEM MCE, do not assess platelet aggregation, therefore platelet aggregometry is needed to assess further detail of platelet function. In our institute, the ROTEM analysis is not covered by the health care insurance, therefore, the study period and number of investigated patients were very limited due to our financial problem. Moreover, the ROTEM analysis was carried out by technicians in the clinical laboratory department and the tests could be done only in day time in the present study due to very limited man power in the clinical laboratory department in the night time. The heterogeneity of patient background, especially regarding operations was the strong limitation of the present study. Nevertheless, we think the present study has a certain meaning in this study field; relationship between coagulation disorder and HCA. Although definitive conclusion may be drawn only from experimental studies, the findings of the present study may contribute to such further studies.

## Conclusion

The volume of perioperative bleeding and intraoperative transfusion of blood products were significantly higher in patients who underwent cardiac surgery with HCA. In ROTEM analysis, MCE platelets were significantly lower in the group with HCA though FIBTEM MCE was similar. ROTEM analysis would indicate that clot firmness contributed by platelet component is reduced in cardiac surgery with HCA.
