# Correction to: Hypothermic circulatory arrest induced coagulopathy: rotational thromboelastometry analysis

**DOI:** 10.1007/s11748-020-01416-0

**Published:** 2020-07-06

**Authors:** Hayato Ise, Hiroto Kitahara, Kyohei Oyama, Keiya Takahashi, Hirotsugu Kanda, Satoshi Fujii, Takayuki Kunisawa, Hiroyuki Kamiya

**Affiliations:** 1 Department of Cardiac Surgery, Asahikawa Medical University, Midorigaoka-Higashi 2-1-1-1, Asahikawa, Hokkaido 078-8510 Japan; 2 Department of Anesthesiology, Asahikawa Medical University, Asahikawa, Hokkaido Japan; 3 Department of Laboratory Medicine, Asahikawa Medical University, Asahikawa, Hokkaido Japan

## Correction to: General Thoracic and Cardiovascular Surgery 10.1007/s11748-020-01399-y

The article “Hypothermic circulatory arrest induced coagulopathy: rotational thromboelastometry analysis”, written by Hayato Ise, Hiroto Kitahara, Kyohei Oyama, Keiya Takahashi, Hirotsugu Kanda, Satoshi Fujii, Takayuki Kunisawa, Hiroyuki Kamiya, was originally published electronically on the publisher’s internet portal on 7 June 2020 without open access. With the author(s)’ decision to opt for Open Choice the copyright of the article changed on 25 June 2020 to © The Author(s) 2020 and the article is forthwith distributed under a Creative Commons Attribution 4.0 International License (https://creativecommons.org/licenses/by/4.0/), which permits use, sharing, adaptation, distribution and reproduction in any medium or format, as long as you give appropriate credit to the original author(s) and the source, provide a link to the Creative Commons licence, and indicate if changes were made.

